# Transport of the uremic toxin symmetric dimethylarginine (SDMA) by renal transport proteins

**DOI:** 10.1007/s00726-025-03466-1

**Published:** 2025-06-25

**Authors:** Lorenz A. Scherpinski, Martin F. Fromm, Renke Maas, Jörg König

**Affiliations:** 1 Institute of Experimental and Clinical Pharmacology and Toxicology, Friedrich-Alexander-Universität Erlangen-Nürnberg, Fahrstr. 17, 91054 Erlangen, Germany; 2 FAU NeW Research Center New Bioactive Compounds, Friedrich-Alexander-Universität Erlangen-Nürnberg, Erlangen, Germany

**Keywords:** SDMA, Uremic toxins, Transport proteins, Arginine, Kidney

## Abstract

The L-arginine derivative and uremic toxin symmetric dimethylarginine (SDMA) is an independent risk marker for total mortality and cardiovascular events. Interferences with L-arginine- or L-homoarginine-related signaling, metabolism, or transport have been proposed as underlying mechanisms. SDMA is endogenously formed and predominantly eliminated via the kidney. Whereas for L-arginine and other L-arginine derivatives such as L-homoarginine and asymmetric dimethylarginine (ADMA) key transport proteins involved in the cellular uptake and release have been characterized, comparable data for the transport of SDMA are lacking.

Using HEK cell lines overexpressing the transport proteins OCT2, OATP4C1, MATE1, OAT4, and OAT10, which are all expressed in renal proximal tubule cells, and the ubiquitously-expressed transport protein CAT1 we performed uptake experiments demonstrating that SDMA is a substrate for CAT1, OATP4C1, OCT2, and MATE1 in physiological concentrations, but not of OAT4 and OAT10. K_m_ values for OATP4C1-, CAT1-, and MATE1-mediated SDMA uptake were 70 µM, 246 µM, and 1 973 µM, respectively. For OCT2-mediated uptake, no saturation could be reached, precluding the determination of a K_m_ value. Uptake of SDMA by these transporters could be inhibited by known substrates of the respective transport proteins. Furthermore, CAT1 and OATP4C1 also mediate the efflux of SDMA out of cells.

These results show that SDMA is a substrate of renally-expressed transport proteins OATP4C1, OCT2, and MATE1 and of CAT1 demonstrating that these transporters are involved in the homeostasis of this uremic toxin and possible sites of interactions with related compounds.

**Supplementary Information:**

The online version contains supplementary material available at 10.1007/s00726-025-03466-1.

## Introduction

Uremic toxins such as the L-arginine derivative symmetric dimethylarginine (SDMA) are substances that accumulate in the plasma of patients with chronic kidney disease (Rosner et al. 2021). SDMA is endogenously formed when proteins containing post-translationally symmetrical dimethylated L-arginine residues are degraded (Lee et al. 1977; Morales et al. 2016). In healthy individuals, the plasma concentration of SDMA is approximately 0.38 to 0.73 µM (Teerlink 2007) but can rise in patients with chronic kidney disease or septic shock up to 2.1 to 6.0 µM (Fleck et al. 2003; Yilmaz et al. 2008; Schwedhelm and Böger 2011; van Wijk et al. 2021). In numerous studies, elevated plasma concentrations of SDMA were found to be an independent risk marker for cardiovascular events and all-cause mortality (Schlesinger et al. 2016). It has been speculated that the connection of SDMA to cardiovascular health relies on a reduction of NO production in endothelial cells caused by competitive inhibition of the transport protein-mediated uptake of L-arginine by SDMA (Bode-Böger et al. 2006; Strobel et al. 2012) and a reduced endothelial nitric oxide synthase (eNOS) stability (Feliers et al. 2015). The increase in SDMA plasma concentrations parallel to the decline of kidney function highlights the kidney as the primary elimination route, with glomerular filtration being the main contributing factor (Kakimoto and Akazawa 1970; Schepers et al. 2009). In patients with normal kidney function, SDMA has a renal clearance of approximately 78.7–90.68 ml/min (Al Banchaabouchi et al. 2000; Gessner et al. 2022) and a protein binding of 7% (Schepers et al. 2014). This indicates a certain net reabsorption (i.e. transporter-mediated uptake) and/or renal metabolism. After uptake into proximal tubule cells, a minor fraction of SDMA can be metabolized by the enzyme alanine–glyoxylate aminotransferase 2 (AGXT2) localized, among others, in proximal tubule cells (Caplin et al. 2012). Furthermore, as SDMA is generated intracellularly in all cells during protein turnover, its release into the bloodstream or, in the case of renal proximal tubule cells, into urine also requires transport mechanisms. So far, the specific transport proteins involved in these processes remain insufficiently characterized. To address this, we focused on solute carrier (SLC) transporters mediating the transport of chemically related molecules, such as L-arginine, asymmetric dimethylarginine (ADMA), and L-homoarginine (Closs et al. 2004; Banjarnahor et al. 2020).

In the context of renal transport, several transporter proteins expressed in the basolateral or luminal membrane of proximal tubule cells have been discussed for their potential role in handling SDMA (Banjarnahor et al. 2020). Among the basolateral transporters, the SLC21/SLCO family member organic anion transporting polypeptide 4C1 (OATP4C1; gene symbol *SLCO4C1*) and the SLC22 family member organic cation transporter 2 (OCT2; gene symbol *SLC22A2*) have received particular attention. OATP4C1 facilitates the renal elimination of uremic toxins, including ADMA (Toyohara et al. 2009; Taghikhani et al. 2019). OCT2 has a broad substrate spectrum, transporting primarily cationic substances including L-arginine (Strobel et al. 2013). In the luminal membrane, the SLC47 family member multidrug and toxin extrusion protein 1 (MATE1; gene symbol *SLC47A1*), and the two SLC22 family members organic anion transporter 4 (OAT4; gene symbol *SLC22A11*) (Ekaratanawong et al. 2004) and OAT10 (gene symbol *SLC22A13*) were investigated. Due to the overlapping substrate spectrum, MATE1 acts together with OCT2 in the secretion of substances (Gessner et al. 2018) and has been characterized as a transporter for L-arginine (Strobel et al. 2013). The substrate spectrum of OAT10 includes several amino acids (Schulz et al. 2014), and OAT4 was studied due to the same subcellular localization and an overlapping substrate spectrum. Another promising candidate for SDMA transport is the widely expressed cationic amino acid transporter 1 (CAT1; gene symbol *SLC7A1*). CAT1 is known to mediate the cellular uptake and the release of L-arginine (Strobel et al. 2012), ADMA (Strobel et al. 2012)d homoarginine (Chafai et al. 2017) and also colocalizes with eNOS in endothelial cells (McDonald et al. 1997).

Since the transport of the uremic toxin SDMA has not been studied in detail, this study aimed to investigate this substance as a substrate of the renally expressed transport proteins CAT1, OATP4C1, OCT2, OAT4, OAT10, and MATE1 using cell models expressing these transport proteins and radioactively labeled SDMA.

## Methods

### Chemicals

[^3^H]-labeled SDMA (molar activity: 1.5 Ci/mmol; specific activity: 5.4 mCi/mg; radiochemical purity by HPLC: 98%) was custom-synthesized by Hartmann Analytic (Braunschweig, Germany). [^3^H]-labeled p-aminohippurate (PAH) (specific activity: 40 Ci/mmol; radiochemical purity by HPLC: 98%) and [^3^H]-labeled estrone-3-sulfate (E3S) (specific activity: 40 Ci/mmol; radiochemical purity by HPLC: 98%) were purchased from American Radiolabeld Chemicals Inc (St Louis). Sodium butyrate and unlabeled substrates L-arginine, ADMA, L-homoarginine, trimethoprim, cimetidine, 1-methyl-4-phenylpyridinium (MPP^+^), and metformin were obtained from Merck (Darmstadt, Germany). Unlabeled SDMA was purchased Medchem (Monmouth Junction, USA). Unlabeled E3S and PAH were obtained from Biomol (Hamburg, Germany).

### Cell culture

HEK-CAT1 (HEK293 cells stably expressing human CAT1) (Strobel et al. 2012), HEK-OATP4C1 (HEK293 cells stably expressing human OATP4C1) (Taghikhani et al. 2019), HEK-OCT2 (HEK293 cells stably expressing human OCT2) (Zolk et al. 2009), HEK-MATE1 (HEK293 cells stably expressing human MATE1) (Müller et al. 2011), and HEK-Co cells [HEK293 cells transfected with the empty expression vector pcDNA3.1(+) or pcDNA3.1/Hygro(−)] were cultured as previously described (Zolk et al. 2009; Müller et al. 2011; Strobel et al. 2012; Taghikhani et al. 2019). Cells were cultured in minimum essential medium supplemented with 10% heat-inactivated fetal bovine serum, 100 U/ml penicillin, 100 µg/ml streptomycin, and either 800 µg/ml geneticin (G418) or 260 µg/ml hygromycin B, in a humidified atmosphere at 37 ^◦^C and 5% CO_2_. Twice a week, cells were subcultured using trypsin (0.05%)-EDTA (0.02%) solution. Growth medium, supplements, and trypsin were obtained by life Technologies GmbH (Darmstadt, Germany).

### Establishment of HEK293 cell lines stably overexpressing human OAT4 or OAT10

The cDNAs of human *SLC22A11* (encoding OAT4) and *SLC22A13* (encoding OAT10) were cloned using human renal total RNA using RT-PCR and the primers oOAT4-RT.for (5`-CAA TGG CCT TTG AGG AGC TCT-3`), oOAT4-RT.rev (5`-GGC ACA ATT TCT AGA GCG AGG-3`) for OAT4 and, oOAT10-RT.for (5`-CAT ACA TGG CTC AGT TTG TCCA-3`), and oOAT10-RT.rev (5`-GTC CAG CTC TTA GAG ACC TCA-3`) for OAT10. The cDNAs were cloned into the pCR2.1-TOPO (Invitrogen GmbH) vector and sequenced. Then, the *SLC22A11* cDNA was subcloned into the pcDNA3.1/Hygro(−) vector (Invitrogen GmbH) and the *SLC22A13* cDNA into the pcDNA3.1(+) vector (Invitrogen GmbH). The resulting plasmids were sequenced and single nucleotide variations leading to amino acid exchanges compared to the reference sequence (*SLC22A11* NM_0184.2 and *SLC22A13* NM_004256.3) were corrected using the QuikChange Multi Site-Directed Mutagenesis Kit (Agilent Technologies Deutschland GmbH, Waldbronn, Germany) resulting in the plasmids pOAT4.31 and pOAT10.31, both containing cDNAs encoding proteins 100% identical with the protein of the reference sequences. Parental HEK293 cells were transfected with the expression vectors using the Effectene™ Transfection Reagent Kit (QIAGEN GmbH, Hilden, Germany). To select OAT4 or OAT10 overexpressing cells, 260 µg/ml hygromycin B and 800 µg/ml geneticin (G418) were used, respectively. The mRNA expression of the transfected HEK-cells was determined using the quantitative real-time PCR (LightCycler^®^ FastStart DNA Master^PLUS^ SYBR Green I ^®^, Roche Diagnostics GmbH Mannheim Germany). The clones with the highest mRNA expression compared to control cells were further characterized by uptake experiments.

### Uptake transport and inhibition assay

HEK-CAT1, HEK-OATP4C1, HEK-OCT2, HEK-MATE1, HEK-OAT4, HEK-OAT10, and HEK-Co cells were seeded in 12-well plates (Sarstedt AG & Co. KG, Nuembrecht, Germany) at a cell density between 5·10^5^ and 7·10^5^ cells/well. After 24 h, protein expression was enhanced by exchanging medium supplemented with 10 mM sodium butyrate (Chen et al. 1997). Uptake experiments were performed 48 h after seeding. Cells were washed with prewarmed (37 °C) uptake buffer (5 mM KCl, 1 mM K_2_HPO_4_, 142 mM NaCl, 1.5 mM CaCl_2_, 1.2 mM MgSO_4_, 5 mM glucose, and 12.5 mM HEPES, pH 7.3 if not stated differently). Radiolabeled SDMA, E3S, PAH, and unlabeled substrates for uptake and inhibition experiments were dissolved in suitable concentrations in uptake buffer. For functional analysis of HEK-OAT4 and HEK-OAT10 the prototypic substrates, E3S and PAH were used, respectively, at an incubation time of 10 min. A pH dependency for OAT10-mediated uptake described in oocytes (Bahn et al. 2008) was further investigated by repeating the PAH uptake at pH 6.0 and 8.0. Initial uptake experiments for all transporters of interest were done using 1 µM SDMA at an incubation time of 1 min. Time dependency was determined to identify the linear phase of the CAT1, OATP4C1, MATE1, and OCT2-mediated transport. For this purpose, cells were incubated with 1 µM SDMA at 1, 2, 5, 10, and 30 min. The kinetic parameters K_m_ values and V_max_ values were determined with SDMA concentrations ranging from 10 to 4 000 µM and an incubation time of 2 min. To investigate inhibitory effects, varying concentrations of transporter-specific inhibitors were added to the uptake buffer along with 1 µM of radiolabeled SDMA and incubated for 2 min. IC_50_ values for the inhibition of CAT1- and OATP4C1-mediated SDMA uptake by L-arginine were determined using L-arginine concentrations between 0.1 and 3 000 µM. After incubation, cells were washed three times with ice-cold uptake buffer and lyzed with 0.2% SDS. Intracellular radioactivity was measured by liquid scintillation counting (TriCarb 2800, Perkin Elmer Life and Analytical Sciences GmbH, Rodgau, Germany). For this, 4 ml of the scintillation solution (Ultima Gold XR; PerkinElmer Life and Analytical Sciences, Inc., Rodgau-Jügesheim, Germany) was added to 500 µl lysate. The protein concentration in each well was determined by bicinchoninic acid assay (BCA Protein Assay Kit, Thermo Fisher Scientific, Bonn, Germany).

### Efflux transport assay

To study CAT1- and OATP4C1-mediated efflux, cells were preincubated with 300 µM SDMA for 60 min. The uptake buffer was removed, and cells were washed once with 1 ml warm (37 ^◦^C) uptake buffer. Then, cells were incubated in 800 µl uptake buffer without SDMA for 2, 5, and 10 min. The transport was stopped by putting the cells on ice. Finally, the supernatant was collected, and cells were washed three times with cold uptake buffer. Radioactivity in the supernatant was determined with liquid scintillation counting. To test for transport protein independent export, efflux experiments were repeated at an incubation temperature of 4 ^◦^C. Baseline values were determined by measuring the radioactivity in the cells directly after preincubation (radioactivity was set to 100%). Before determining intracellular radioactivity, cells were washed once with 1 ml warm uptake buffer to remove radioactivity bound to the plasma membrane and then washed three times with ice-cold uptake buffer.

### Data analysis and statistics

Net transport was calculated as the difference between the uptake into HEK-Co control cells and uptake into HEK-CAT1, HEK-OATP4C1, HEK-MATE1, and HEK-OCT2 cells, respectively. Transport data were normalized to the protein value of the respective well. All data points are presented as means ± SEM with at least six biological replicates. K_m_ and V_max_ values and their respective 95% confidence intervals were calculated with a single nonlinear regression curve fit through the data points using Michaelis-Menten enzyme kinetics in GraphPad Prism (Version 5.01, 2007, GraphPad Software, San Diego, CA, USA). For inhibition experiments and the determination of the IC_50_ values, the apparent net SDMA uptake without added inhibitor was set to 100%. IC_50_ values and their 95% confidence intervals were calculated using a single dose-response fit through the data points in GraphPad Prism with the implemented formula [Y = Bottom + (Top-Bottom) / 1 + 10^(X-LogIC_50_)]. Statistical significance was analyzed with an unpaired two-tailed t-test and multiple comparisons by one-way ANOVA with subsequent Tukey-Kramer multiple comparison test using GraphPad Prism. A *p*-value of less than 0.05 was considered significant.

## Results

### Characterization of newly-established OAT4- and OAT10-overexpressing HEK293 cells

Whereas HEK cell lines stably expressing CAT1, OATP4C1, MATE1, and OCT2 have already been described (Zolk et al. 2009; Müller et al. 2011; Strobel et al. 2012; Taghikhani et al. 2019), OAT4- and OAT10-expressing cell lines were established during this project. The functionality of these newly-established HEK-OAT4 and HEK-OAT10 cell lines was analyzed using the prototypic substrate E3S for OAT4 and PAH for OAT10, demonstrating uptake values of 14.1-fold for HEK-OAT4 (Supplementary Fig. 1a) and 3.3-fold for HEK-OAT10 (Supplementary Fig. 1b) compared to the uptake into HEK-Co control cells. In addition, a known pH-dependency for OAT10 could be shown (Supplementary Fig. 1b).

### Initial uptake experiments with SDMA as a substrate

**Fig. 1:**
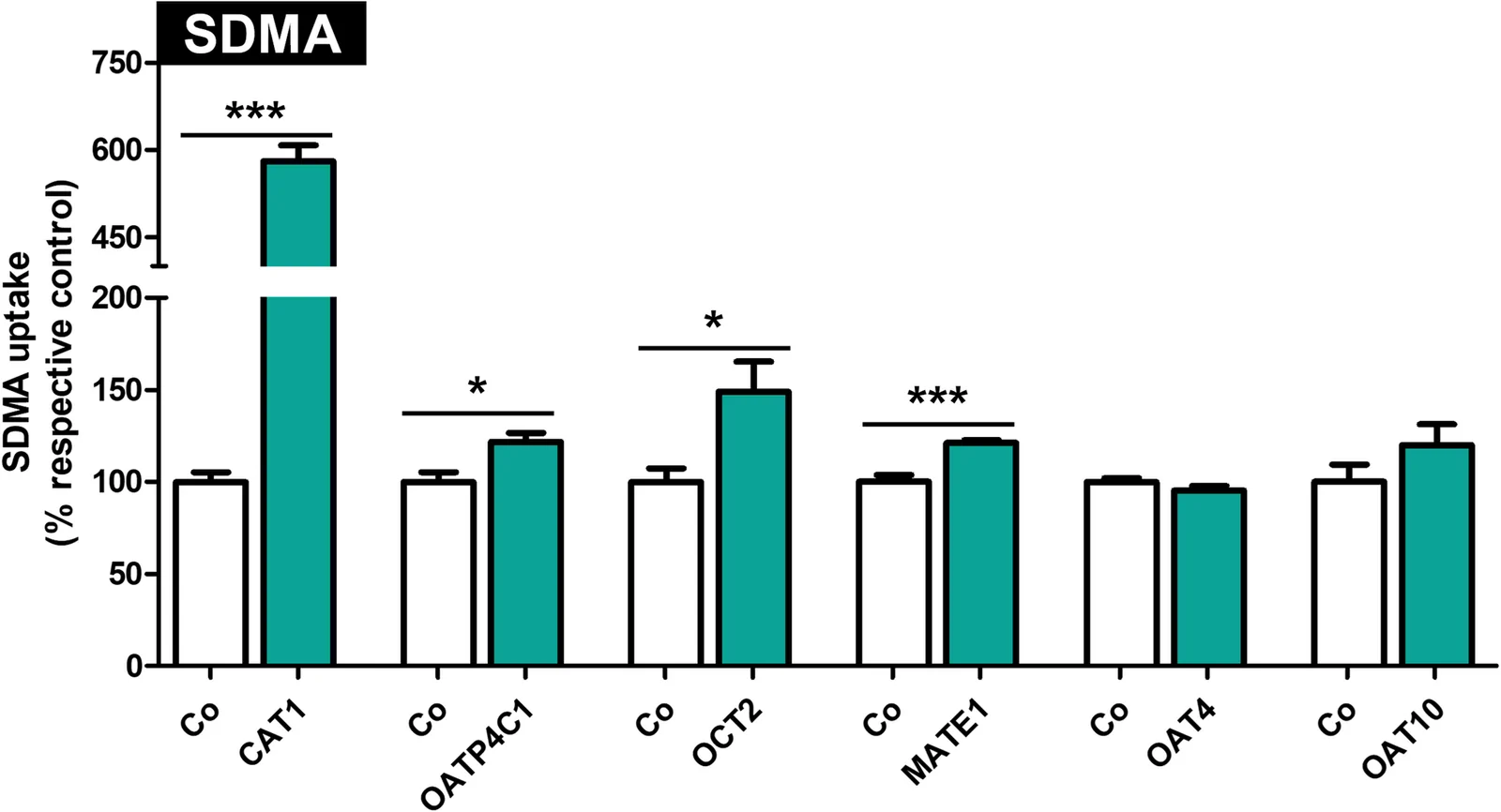
Initial uptake experiments using 1 µM SDMA as a substrate in respective HEK293 cell lines expressing different transport proteins. Uptake into control cells was set to 100%. White bars– uptake into HEK-Co control cells, green bars– uptake into transporter expressing cells. Uptake time was 1 min. Experiments were conducted with six biological replicates (i.e., *n* = 6). Data are shown as mean ± SEM (standard error of the mean). Statistical analysis was performed using an unpaired two-tailed t-test. *p < 0.05, ***p < 0.001 vs. uptake into control cells.

To study SDMA as a substrate for all transporters, initial uptake experiments with 1 µM SDMA (i.e., in its physiological range) were conducted. As shown in Fig. 1, significant uptake was observed for CAT1-, OATP4C1-, OCT2-, and MATE1-mediated transport. Since no significant uptake could be detected using HEK-OAT4 and HEK-OAT10 cells, even when studied under different uptake conditions using known cosubstrates (data not shown), we assume that SDMA is not a relevant substrate of OAT4 and OAT10. Therefore, OAT4 and OAT10 were not further characterized.

### Time-dependency of SDMA uptake

**Fig. 2:**
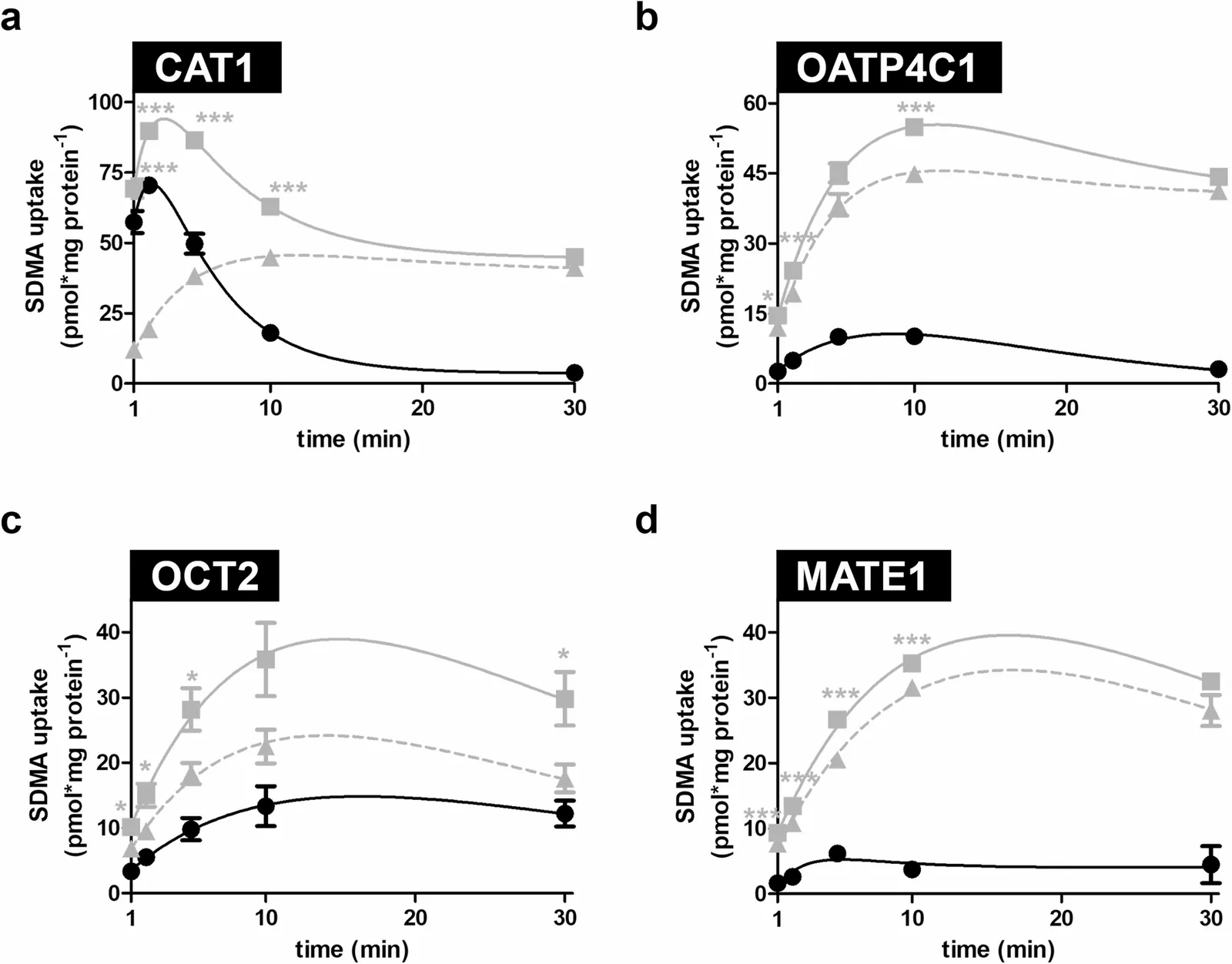
Time-dependent uptake of SDMA (1 µM). Time-dependency was measured using (**a**) HEK-CAT1 cells, (**b**) HEK-OATP4C1 cells, (**c**) HEK-OCT2 cells, (**d**) HEK-MATE1 cells, and the respective HEK-Co control cells. Net uptake was calculated by subtracting the uptake into control cells from the uptake into transporter-overexpressing cells. Uptake was measured for 1, 2, 5, 10, and 30 min. Experiments were conducted with six biological replicates (i.e., *n* = 6). Data are shown as mean ± SEM (standard error of the mean). Statistical analysis was performed using an unpaired two-tailed t-test. *p < 0.05, **p < 0.01, ***p < 0.001 vs. uptake into control cells. ■ = Uptake into transporter expressing cells; ▲ = Uptake into HEK-Co cells; ● = Net uptake.

Time-dependent uptake experiments were performed using HEK-CAT1, HEK-OATP4C1, HEK-OCT2, HEK-MATE1, and respective HEK-Co control cells to determine optimal conditions for the subsequent transport and inhibition studies. These results demonstrate that SDMA (1 µM) was significantly taken up by HEK-CAT1 (Fig. 2a), HEK-OATP4C1 (Fig. 2b), HEK-OCT2 (Fig. 2c), and HEK-MATE1 (Fig. 2d) cells compared to the uptake into the respective control cells. For all transporters, linear uptake could be detected within the first two minutes. Therefore, an incubation time of two minutes was used for all further transport experiments.

### Kinetic transport constants for CAT1-, OATP4C1-, OCT2-, and MATE1-mediated SDMA uptake

**Fig. 3:**
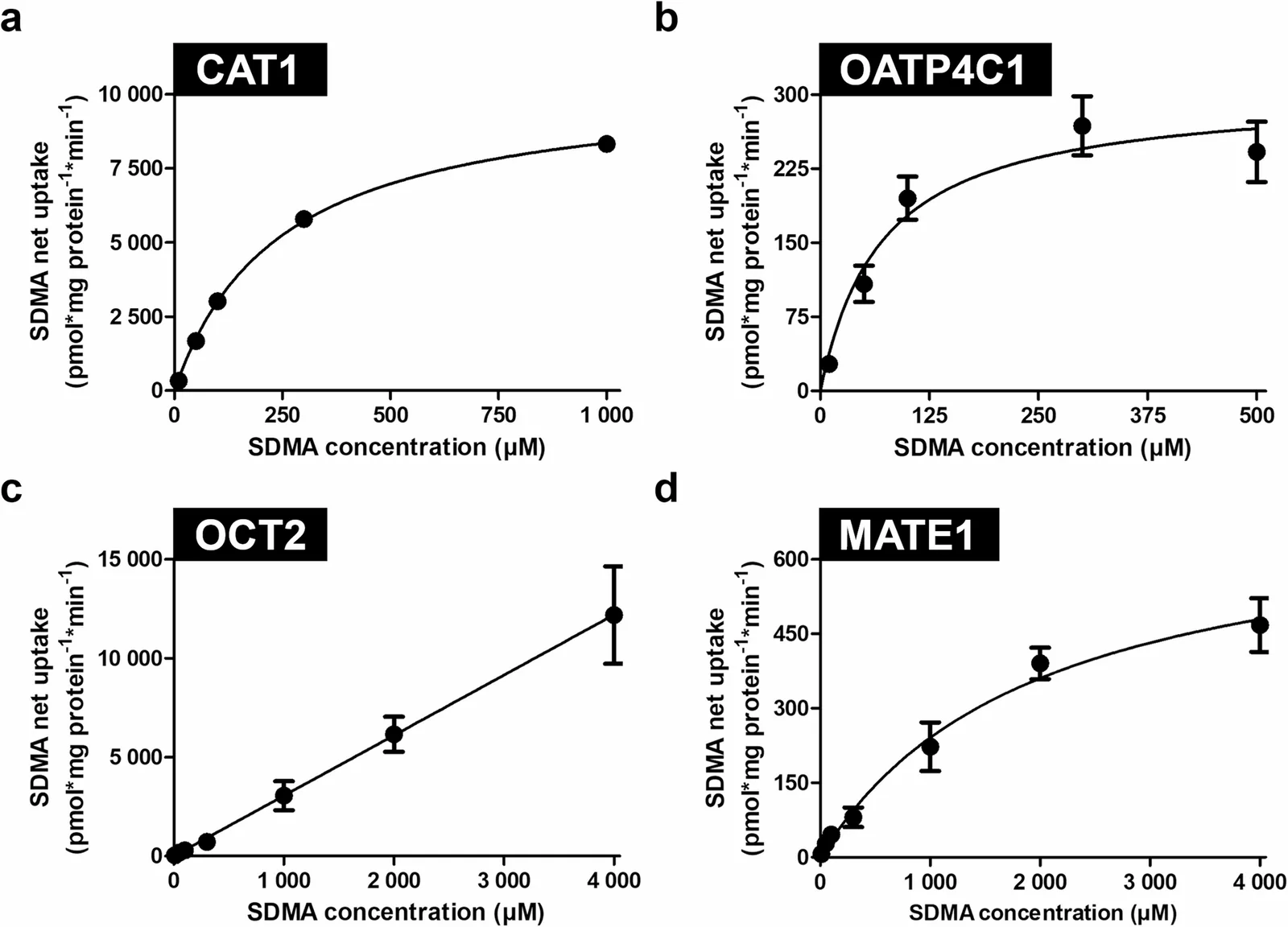
Determination of kinetic transport constants (K_m_ values) and maximal transport velocities (V_max_ values) for transporter-mediated SDMA uptake. Shown are net uptake values for SDMA transport in (**a**) CAT1-, (**b**) OATP4C1-, (**c**) OCT2-, and (**d**) MATE1-overexpressing HEK cells. Uptake was measured after 2 min. Experiments were conducted with six biological replicates (i.e., *n* = 6). Data are shown as mean ± SEM (standard error of the mean). The error bars in (a) are in size of the symbols. K_m_ and V_max_ values and their respective 95% confidence intervals were determined using nonlinear regression with Michaelis-Menten enzyme kinetics curve fit.

The kinetic transport constants (K_m_ and V_max_ values) were determined using standardized uptake conditions at varying SDMA concentrations and the established incubation time. Whereas for CAT1-, OATP4C1-, and MATE1-mediated uptake, saturation could be reached (Fig. 3a, b, d, and Table 1). No saturation of SDMA transport could be detected even at the highest used and feasible concentration of 4 000 µM for OCT2 (Fig. 3c; Table 1).

**Table 1:** Kinetic transport constants K_m_ and V_max_ for transporter-mediated SDMA transport

Transporter	K_m_ in µM	V_max_ in pmol*mg protein^− 1^*min^− 1^
CAT1 (*SLC7A1*)	246 (95% CI: 214–279)	10 412 (95% CI: 9 895–10 929)
OATP4C1 (*SLCO4C1*)	70 (95% CI: 21–121)	303 (95% CI: 241–365)
OCT2 (*SLC22A2*)	No saturation	No saturation
MATE1 (*SLC47A1*)	1 973 (95% CI: 640–3 305)	716 (95% CI: 493–939)

### Influence of other substrates on transporter-mediated SDMA uptake

**Fig. 4:**
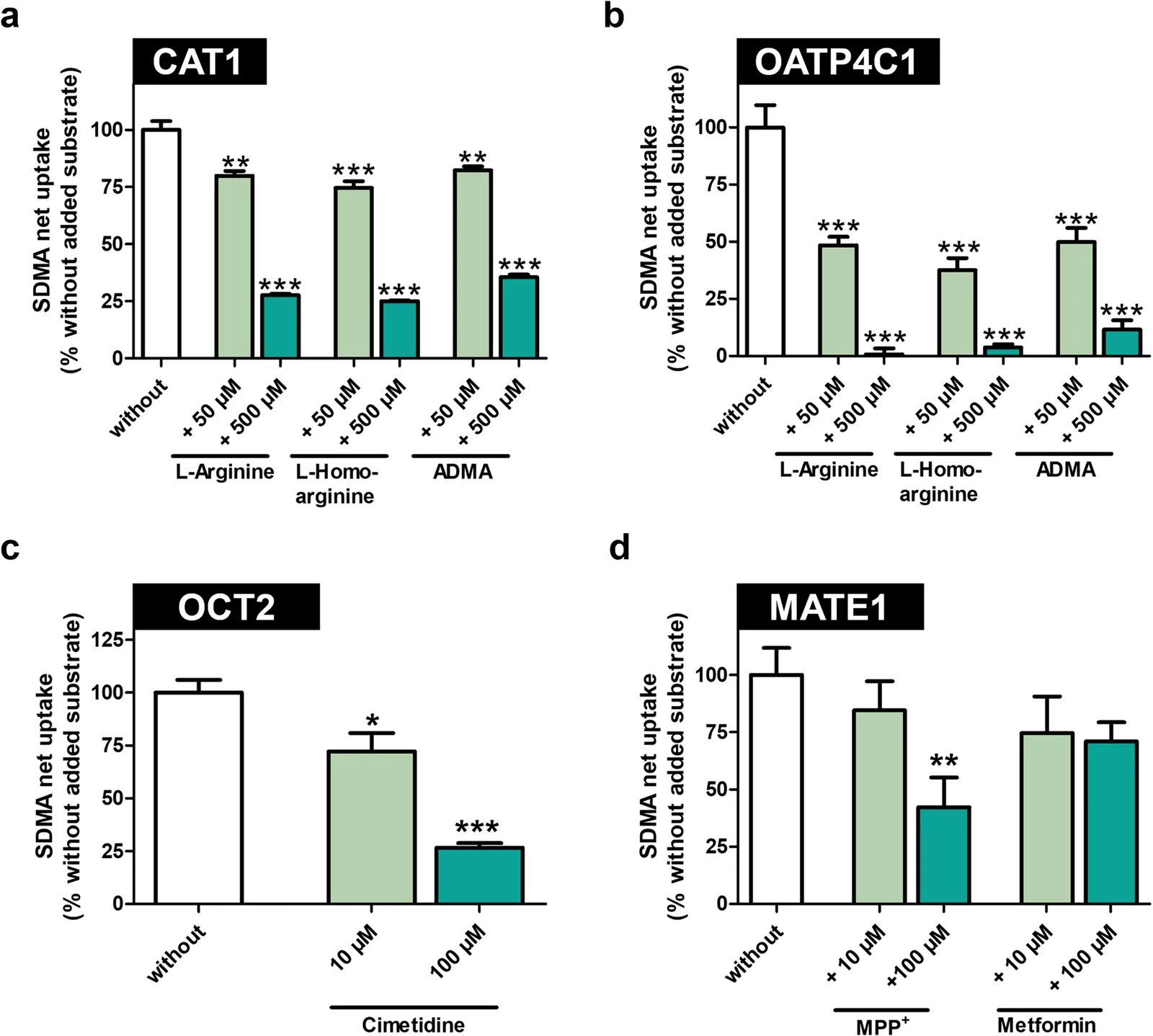
Influence of known transporter substrates and inhibitors on SDMA uptake. (**a**) In HEK-CAT1 cells and (**b**) HEK-OATP4C1 cells, SDMA uptake was reduced by 50 µM (light green) or 500 µM (dark green) L-arginine, L-homoarginine, or ADMA. (**c**) OCT2-mediated SDMA uptake was reduced by 10 µM (light green) or 100 µM (dark green) of cimetidine. (**d**) MATE1-mediated SDMA uptake was reduced by 10 µM (light green) or 100 µM (dark green) of MPP^+^ or metformin. Net uptake without added substances is set to 100% (white). Uptake was measured after 2 min. Experiments were conducted with six biological replicates (i.e., *n* = 6). Data are shown as mean ± SEM (standard error of the mean). Statistical analysis was performed using an unpaired two-tailed t-test. *p < 0.05, ** *p* < 0.01 and *** *p* < 0.001 versus uptake without added substance.

**Fig. 5:**
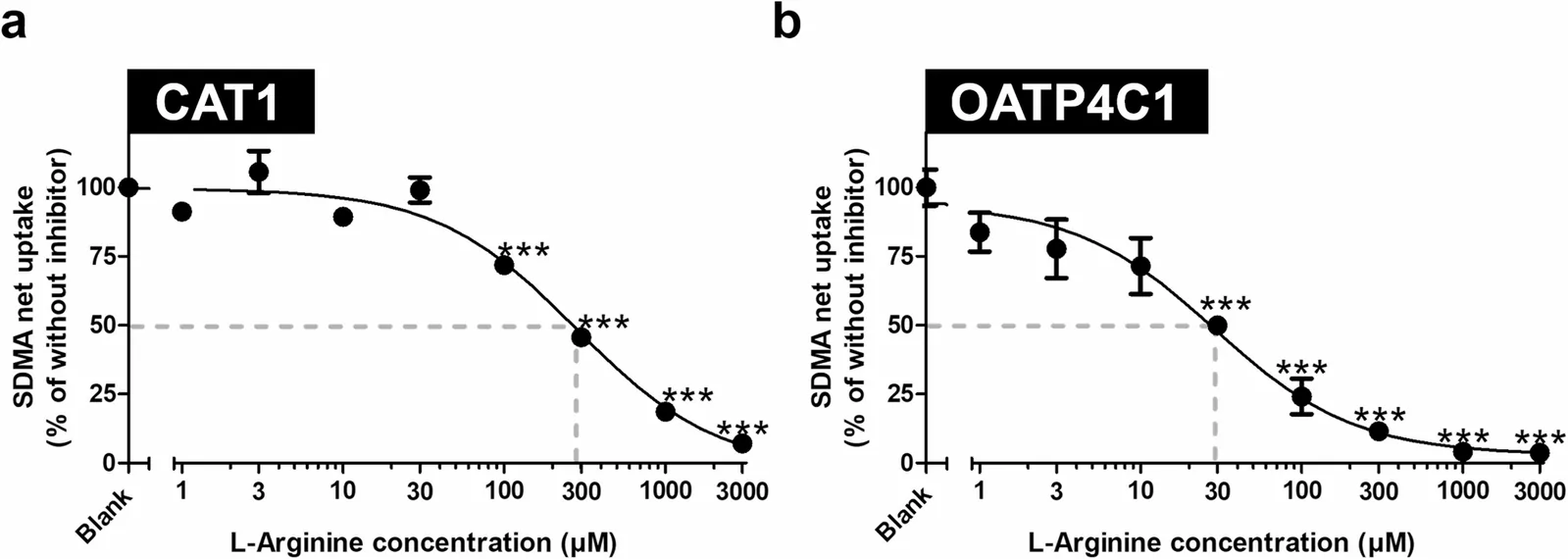
IC_50_ values for the inhibition of (**a**) CAT1- and (**b**) OATP4C1-mediated SDMA uptake by L-arginine. The net uptake of SDMA is given in relation to the net uptake without added L-arginine (100%). Uptake was measured after 2 min. Experiments were conducted with six biological replicates (i.e., *n* = 6). Data are shown as mean ± SEM (standard error of the mean). IC_50_ values were determined using a nonlinear fit log(inhibitor) vs. response. Statistical analysis was performed using one-way ANOVA with subsequent Tukey-Kramer multiple comparison test. ***p *<* 0.001 vs. uptake without added arginine.

Next, we investigated if known substrates of these four transporters affect SDMA uptake. L-arginine, L-homoarginine, and ADMA have been characterized as substrates for both CAT1 and OATP4C1 (Strobel et al. 2012; Taghikhani et al. 2019) and all influenced SDMA transport, thereby competing with SDMA for CAT1- or OATP4C1-mediated uptake (Fig. 4a + b). OCT2-mediated SDMA transport was significantly reduced by 10 µM and 100 µM of the prototypic inhibitor cimetidine (Fig. 4c). For MATE1, the known substrates MPP^+^ and metformin were used, demonstrating for MPP^+^ (100 µM) a significant reduction of MATE1-mediated SDMA uptake (Fig. 4d). A slight but not significant reduction by 30% could be observed for 100 µM metformin (Fig. 4d). Furthermore, IC_50_ values for the inhibition of CAT1- and OATP4C1-mediated SDMA uptake by L-arginine were determined (Fig. 5; Table 2). These experiments demonstrated that OATP4C1-mediated SDMA uptake was inhibited with an IC_50_ value of 28.8 µM (Fig. 5b; Table 2), whereas for CAT1 the IC_50_ value is approximately 10-fold higher with 280.0 µM (Fig. 5a; Table 2).

**Table 2:** Parameters of the nonlinear regression analysis for the determination of the IC_50_ value for the inhibition of CAT1- and OATP4C1-mediated SDMA uptake by L-arginine

Parameters	CAT1	OATP4C1
IC_50_	280 µM (95% CI: 208–377 µM)	28.8 µM (95% CI: 17.10–48.7 µM)
Min	-2.2% (95% CI: -8.9–4.4%)	3.0% (95% CI: -4.2–10.3%)
Max	99.66% (95% CI: 96.5–102.8%)	94.0% (95% CI: 86.7–101.3%)

### CAT1- and OATP4C1-mediated efflux of SDMA

**Fig. 6:**
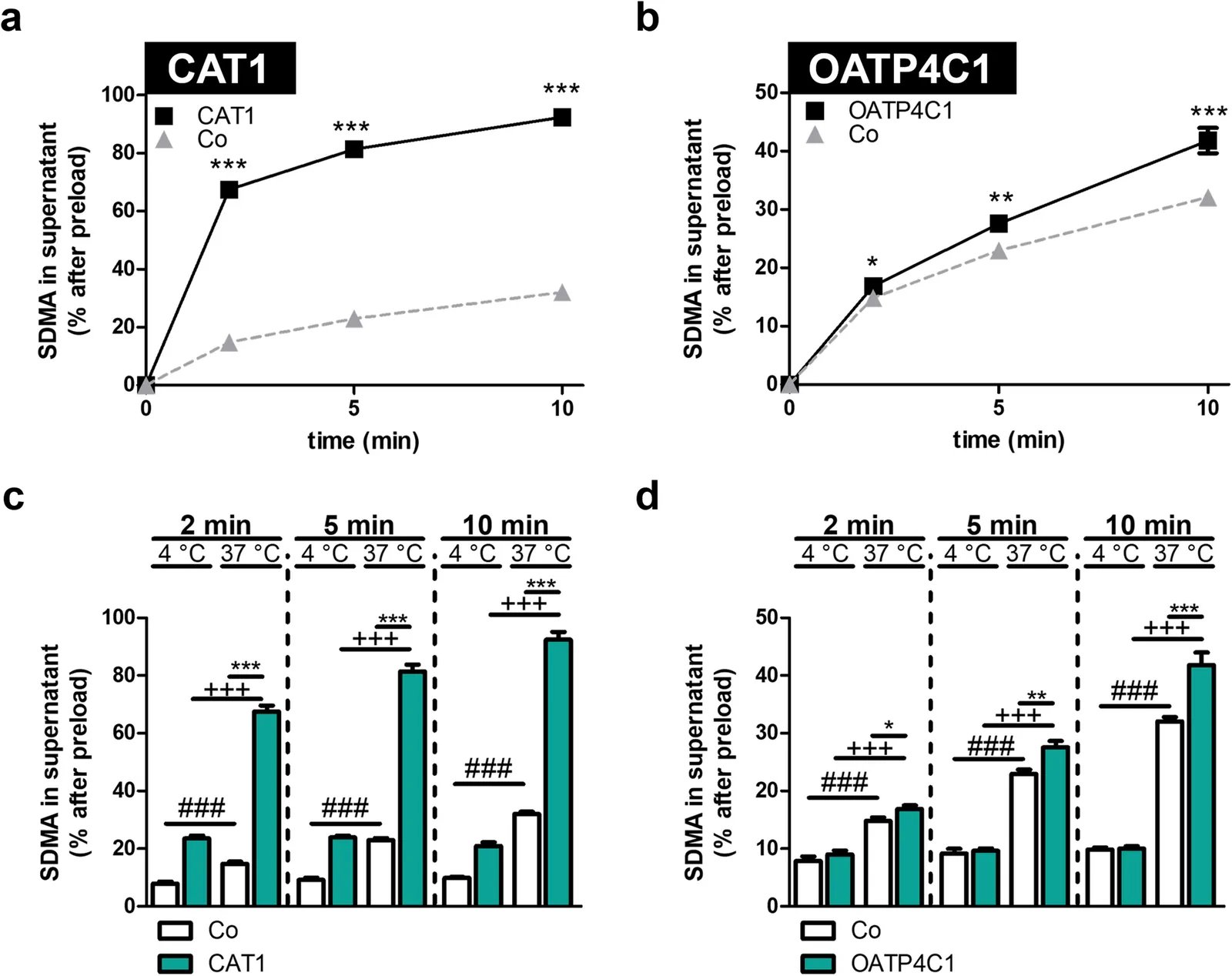
CAT1- and OATP4C1-mediated efflux of SDMA. (**a**) HEK-CAT1, (**b**) HEK-OATP4C1, and HEK-Co cells were preloaded with 300 µM SDMA for one hour, and the efflux of SDMA over time into the supernatant for the transporter overexpressing cells (■) and the control cells (▲) was determined. The intracellular radioactivity after preloading was set to 100%, and the values are given related to this concentration. Temperature-dependency of the transporter-mediated SDMA efflux for (**c**) HEK-CAT1 and (**d**) HEK-OATP4C1. Experiments were conducted with six biological replicates (i.e., *n* = 6). Data are shown as mean ± SEM (standard error of the mean). The error bars in (**a**) are in size of the symbols. Statistical analysis was performed using an unpaired two-tailed t-test. *p *<* 0.05; **p *<* 0.01 and ***p *<* 0.001 efflux of overexpressing cells at 37 °C vs. efflux of control cells at 37 °C, +*p* *<* 0.05; ++*p* *<* 0.01 and +++*p* *<* 0.001 efflux of overexpressing cells at 37 °C vs. efflux of overexpressing cells at 4 °C, #*p* *<* 0.05; ##*p* *<* 0.01 and ###*p* *<* 0.001 efflux of control cells at 37 °C vs. efflux of control cells at 4 °C.

Since it was demonstrated that CAT1 and OATP4C1 can also mediate efflux of substrates (Strobel et al. 2012; Taghikhani et al. 2019), we finally investigated if both transporters also mediate SDMA efflux. After preloading the cells, we detected significantly more SDMA in the supernatant of HEK-CAT1 (Fig. 6a) and HEK-OATP4C1 transfectants (Fig. 6b) compared to the supernatant of the respective control cells. Furthermore, time-dependent efflux experiments at 4 °C and 37 °C were performed to confirm that this efflux is transporter-mediated. These results are in line with the initial efflux experiments using other substrates and demonstrated protein-mediated efflux of SDMA for both CAT1 (Fig. 6c) and OATP4C1 (Fig. 6d).

## Discussion

SDMA originates from the proteolytic breakdown of proteins and is classified as uremic toxin (Lee et al. 1977; Morales et al. 2016). It is an independent risk marker for cardiovascular events and total mortality (Schlesinger et al. 2016). Data regarding transport proteins involved in the handling of SDMA are lacking. Therefore, we used custom-synthesized, radiolabeled SDMA for uptake experiments in HEK293 cell lines recombinantly overexpressing renally expressed transport proteins and were able to determine highly significant net uptake mediated by CAT1, OATP4C1, OCT2, and MATE1 (Fig. 1). Uptake could be detected at the lowest SDMA concentration tested (1µM), which is around the plasma concentration of SDMA in healthy subjects, indicating their likely involvement in the cellular uptake of SDMA under physiological conditions (Fleck et al. 2003; Yilmaz et al. 2008; Schwedhelm and Böger 2011). In contrast, no transporter-mediated SDMA uptake was observed for OAT4 or OAT10 (Fig. 1) at this concentration. Since the mRNA expression and transport experiments with respective standard substrates confirmed functionality of OAT4 and OAT10 in our cell lines (Supplementary Fig. 1a and 1b), we found that SDMA is not a substrate of these two transporters under our experimental conditions. Nevertheless, it has been shown that OAT4- and OAT10-mediated transport may depend on co-substrates (Ekaratanawong et al. 2004; Bahn et al. 2008), and it is possible that a required co-substrate was not present in the uptake assay. The other transporters were further characterized, and we calculated apparent K_m_ values of 246, 70, and 1 973 µM for cellular uptake of SDMA mediated by CAT1, OATP4C1, and MATE1, respectively (Fig. 3a-c; Table 1). No saturation in the range of tested concentrations could be detected for OCT2 (Fig. 3c; Table 1). The K_m_ value for the uptake of SDMA by CAT1 is very similar to the K_m_ value of the structurally related substrates ADMA (Strobel et al. 2012) or L-homoarginine (Chafai et al. 2017) (Table 3), which were previously determined by our group in a comparable experimental setting. As shown for L-arginine and ADMA, MATE1 has a low affinity and low transport capacity for SDMA, whereas OCT2 shows a low affinity but high capacity for SDMA (Strobel et al. 2013).

**Table 3:** Comparison of cellular uptake kinetics of SDMA to L-arginine, L-homoarginine, and ADMA. n.d.: K_m_ values were not determined; SDMA: symmetric dimethylarginine; ADMA: asymmetric dimethylarginine

Transporter	K_m_ SDMA (µM)	K_m_ ADMA (µM)	K_m_ L-arginine (µM)	K_m_ L-homoarginine (µM)
CAT1 (*SLC7A1*)	246.0	183.0 (Strobel et al. 2012)	519.0 (Strobel et al. 2012)	175.0 (Chafai et al. 2017)
OATP4C1 (*SLCO4C1*)	70.0	232.1 (Taghikhani et al. 2019)	48.1 (Taghikhani et al. 2019)	49.9 (Taghikhani et al. 2019)
OCT2 (*SLC22A2*)	No saturation	967.0 (Strobel et al. 2013)	> 10 000 (Strobel et al. 2013)	n.d.
MATE1 (*SLC47A1*)	1 973.0	n.d.	n.d.	n.d.

All K_m_ values are substantially above the reported plasma concentrations of SDMA, ranging from 0.38 to 0.73 µM in healthy subjects (Teerlink 2007) and can reach up to 6 µM in conditions such as septic shock or chronic kidney disease (van Wijk et al. 2021). The renal net clearance of SDMA is primarily driven by glomerular filtration. However, transport proteins contribute by facilitating uptake and renal metabolism. That the investigated transport proteins could influence the SDMA homeostasis in vivo is supported by the observation that even at high concentrations of known inhibitors, complete inhibition of SDMA transport was not achieved. For CAT1 and OATP4C1, the reported K_m_ values of L-arginine were in the range of physiological L-arginine concentrations (Au et al. 2023). Therefore, we determined IC_50_ values for L-arginine inhibition of CAT1- and OATP4C1-mediated SDMA uptake. Both IC_50_ values suggested that under physiological conditions, L-arginine is competing with SDMA for both transporters. Considering their bidirectional character, we also conducted efflux experiments for CAT1 and OATP4C1. As demonstrated for ADMA in previous studies, both transporters showed the capability to mediate the efflux of SDMA, demonstrating the importance of CAT1 and OATP4C1 for the overall homeostasis of these substances (Strobel et al. 2012; Taghikhani et al. 2019).

CAT1 co-localizes with eNOS in endothelial cells, which metabolize L-arginine to NO. As part of the “arginine-paradox”, it has been speculated that SDMA could compete with L-arginine uptake, thereby lowering the cellular supply of L-arginine. However, the low affinity of SDMA to CAT1 makes it unlikely that, under physiological conditions, SDMA significantly affects L-arginine uptake. On the other hand, CAT1 is almost ubiquitously expressed and could be one key efflux mechanism as the first step in the cellular elimination of SDMA, which is produced in every cell. Under the aspect of renal homeostasis, gene expression data indicate that CAT1 expression in the kidney is primarily restricted to cells of the collecting duct and not in proximal tubule cells, where amino acid homeostasis occurs (Cheval et al. 2012). This suggests that CAT1 is more involved in the overall homeostasis of these substances rather than in their renal handling.

It has been shown that transgenic rats overexpressing human OATP4C1 had significantly lower ADMA plasma concentrations and less pronounced hypertension in the setting of renal failure (Toyohara et al. 2009). Since the physiological plasma concentration of ADMA is in a similar range to that of SDMA, and the K_m_ value for SDMA is three times lower than the K_m_ value of ADMA for OATP4C1-mediated transport, this could indicate a physiological relevance for OATP4C1-mediated SDMA uptake. However, the calculated K_m_ value overlaps more with the K_m_ value of L-homoarginine, for which the OATP4C1-mediated efflux out of the proximal tubule has previously been proposed (Taghikhani et al. 2019). Which transport direction dominates under physiological conditions may depend on both extracellular and intracellular substrate concentrations and should be investigated in more detail.

OCT2 and MATE1 were investigated due to their broad substrate spectrum of cationic xenobiotics and endogenous substances, including several uremic toxins (Otsuka et al. 2005; Tanihara et al. 2007; Giacomini et al. 2010; Schophuizen et al. 2013). Based on their coordinate transport and their role in the renal elimination of widely prescribed drugs, Galetin et al. categorized OCT2 and MATE1 as clinically important transporters of category A that should always be considered during drug development (Galetin et al. 2024). The fact that no saturation for OCT2-mediated SDMA uptake could be reached indicates a very low affinity for SDMA, suggesting that SDMA is not a major substrate for this transporter. However, the partial reduction in transport observed for both OCT2 and MATE1 (Fig. 4c + d) suggests that they may still play a role in the homeostasis of SDMA. MATE1-mediated SDMA transport has been investigated using uptake experiments, demonstrating that SDMA is a low-affinity substrate for MATE1. Under physiological conditions, MATE1 functions as an efflux transporter, driven by an H⁺ gradient which facilitates the export of substrates from proximal tubule cells into urine (Otsuka et al. 2005). To study MATE1-mediated drug transport through uptake experiments, this gradient is reversed, turning MATE1 into an uptake transporter as recommended by the EMA (European Medicines Agency 2024). Consequently, as observed for other substrates, the affinity of SDMA for MATE1-mediated export may differ from the determined K_m_ value (Müller et al. 2011). Therefore, the role of MATE1 in the export of substances into urine should be further investigated. A possible link of OCT2-MATE1-mediated transport of L-arginine and L-arginine derivatives was described by Aleidi et al. (Aleidi et al. 2020). In their study, they found that patients in the obese type 2 diabetes mellitus group treated with metformin had altered L-arginine and L-homoarginine levels (Aleidi et al. 2020). Since metformin is a substrate of both OCT2 and MATE1 and metformin was able to reduce the MATE1-mediated SDMA uptake in vitro, it could be possible that SDMA levels were also altered in this patient group.

In summary, we could demonstrate that SDMA is a substrate of the renally expressed transport proteins OCT2, OATP4C1, and MATE1 and of the ubiquitously expressed transporter CAT1. Together with previous studies, uptake and inhibition experiments demonstrated that the investigated transporters are involved in the homeostasis of L-arginine and of cardioactive L-arginine derivatives such as SDMA.

## Electronic supplementary material

Below is the link to the electronic supplementary material.

Supplementary Material 1

## Data Availability

The data presented in this study are available from the corresponding author upon reasonable request.
