# Supplementary material for: Transport of the uremic toxin symmetric dimethylarginine (SDMA) by renal transport proteins
Source: Amino Acids. 2025 Jun 25;57:34. doi: 10.1007/s00726-025-03466-1 (PMC12187869; doi:10.1007/s00726-025-03466-1)
Supplement: Supplementary file 1 [file 726_2025_3466_MOESM1_ESM.docx]

Supplementary Informations

**Transport of the uremic toxin symmetric dimethylarginine (SDMA) by**

**renal transport proteins**

Lorenz A. Scherpinski^1^, Martin F. Fromm^1,2^, Renke Maas^1,2^ & Jörg König^1,2^

^1^Institute of Experimental and Clinical Pharmacology and Toxicology, Friedrich-Alexander-Universität Erlangen-Nürnberg, Erlangen, Germany

^2^FAU NeW Research Center New Bioactive Compounds, Friedrich-Alexander-Universität Erlangen-Nürnberg, Erlangen, Germany

Address for correspondence: Prof. Jörg König, Institute of Experimental and Clinical Pharmacology and Toxicology, Friedrich-Alexander-Universität Erlangen-Nürnberg, Fahrstr. 17, 91054 Erlangen, Germany. Tel: +49-9131-8522077; E-mail: joerg.koenig@fau.de;

**Supplementary Fig 1** Functional characterization of newly established HEK-OAT4 and HEK-OAT10 cells. Functional characterization of (**a**) HEK-OAT4 and (**b**) HEK-OAT10 using 1 µM estrone-3-sulfate (E3S) and 1 µM p-aminohippurate (PAH), respectively. HEK-OAT10-mediated uptake was further investigated at different pH values.
